# Non-*Helicobacter pylori Helicobacters*, a Treatable Provocateur of Parkinson’s Disease: Hypothesis, Evidence and Species Specificity

**DOI:** 10.3390/ijms252313123

**Published:** 2024-12-06

**Authors:** Wenjing Wang, Melvyn Smith, Richard Ellis, Antonella Savio, Amanda Nevel, Chianna Umamahesan, Polychronis Pavlidis, Bu’ Hussain Hayee, David Taylor, Allan H. Young, André Charlett, Sylvia M. Dobbs, R. John Dobbs

**Affiliations:** 1 Host Microbiome Interaction, Clinical Pharmacology and Therapeutics, Institute of Pharmaceutical Science, King’s College London, London SE1 9NH, UK; 2 South London and Maudsley NHS Foundation Trust, London SE5 8AB, UK; 3 Institute of Psychiatry, Psychology & Neuroscience, King’s College London, London SE5 8AF, UK; 4 Department of Infectious Diseases, King’s College Hospital, London SE5 9RS, UK; 5 Animal and Plant Health Agency, Weybridge KT15 3NB, UK; 6 Agriculture and Horticulture Development Board (AHDB-Pork), Coventry CV3 4PE, UK; 7 Department of Gastroenterology, King’s College Hospital, London SE5 9RS, UK; 8 Statistics, Modelling and Economics, UK Health Security Agency, London NW9 5EQ, UK

**Keywords:** Parkinson’s disease, mortality, depression, farmers, non-*H. pylori Helicobacters*, *H. pylori*, *H. suis*, systematic review

## Abstract

Epidemiological and eradication trial evidence indicates that *Helicobacter pylori*, a major causative factor in peptic ulcer and gastric cancer, is a driver of the hypokinesia of Parkinson’s disease (PD). Psychological (cognitive impairment, depression and anxiety) and gastrointestinal (peptic ulceration and constipation) PD features can precede the symptomatic onset of motor features by decades. We hypothesise that the non-*H. pylori Helicobacters* (NHPH), which have farm, companion and wild animals as their main hosts, can have a role in PD aetiopathogenesis. In those occupationally at risk of NHPH infection, we address whether there is increased mortality with PD, or depression or suicide. Our systematic review gave evidence that occupational exposure to animals/their products is associated with excess mortality with PD. Indeed, whilst livestock farming increased the risk, crop farming decreased it. Moreover, excess mortality from non-Hodgkin lymphoma in livestock farmers is compatible with NHPH being causal. Our scoping review showed that farmers, veterinarians and abattoir workers have an increased risk of depression and suicide; whether their depression is associated with being down the pathway to PD and/or the presence of *Helicobacter* infection needs investigation. Regarding *Helicobacter* species specificity, the link between the presence of NHPH in gastric biopsy and PD was described using a *ureA* polymerase chain reaction (PCR) assay, targeting the most-commonly named NHPH, *H. suis*. We describe its redesign and optimisation as a probe-based PCR, confirming the exclusion of *H. pylori* but not *H. suis* specificity (additionally identifying 6 species of a 22-NHPH-species panel). The exploration of the zoonotic hypothesis requires a non-invasive pan-*Helicobacter* PCR screen, allowing the detection and molecular grouping of *Helicobacter* species.

## 1. Introduction

Our 2020 systematic review [1] confirmed the importance of *Helicobacter pylori*, the usual species of the genus *Helicobacter* infecting the human stomach, as a driver of the hypokinesia of Parkinson’s disease (PD). The increase in PD diagnosis with a history of *Helicobacter* infection has been demonstrated in two nationwide (Denmark and Taiwan) studies [2,3]. There is a birth cohort effect for the serum concentration of anti-urease-IgG-antibody (i.e., titre increased with age) in controls, but this effect was absent in PD [4]. Those with PD were twice as likely to be seropositive before the age of 72.5 years. This differential age trend (not attributable to social class) is compatible with *Helicobacter* being a causal factor for PD. Moreover, a randomised placebo-controlled trial showed that successful (biopsy-proven) *H. pylori* eradication in PD is disease-modifying [5,6], even in anti-Parkinsonian treatment-naïve patients [7]. Hypokinesia regresses following *H. pylori* eradication, whilst failed eradication, including persistence only at the polymerase chain reaction (PCR) level of detection, increases hypokinesia [6]. Autoimmunity, or pattern recognition at higher taxonomic levels, may underlie the benefit of eradicating low-density *Helicobacter* infection and the adverse consequence of its persistence. However, the antimicrobials given, or the *H. pylori* eradication itself, appear to unlock the next stage in the natural history, the development of rigidity [6]. That is eliminating one aetiopathogenic driver might allow another to come to the fore. The subsequent driver may be intestinal dysbiosis, or *Helicobacter* strains or species with different antimicrobial sensitivities or previously suppressed by *H. pylori* [8,9].

The ‘non-*H. pylori Helicobacters*’ (NHPH) have farm, companion or wild animals as their main host. Some NHPH species are known to infect humans, the most frequently named being *H. suis*, which has a substantial reservoir in livestock pigs, in the UK [10] and worldwide. We have reported biopsy-proven eradication of a characteristically corkscrew-shaped NHPH in a urea breath-test-positive PD patient with antral gastritis [11]. In this cachectic, previously wheelchair-bound patient, there was a corresponding U-turn in brady/hypokinesia, failing computing skills and a decreasing body mass index. Our collaborators at Ghent University designed a PCR assay to detect *H. suis* in gastric biopsy DNA extract, based on the *ureA* gene with complementary amplicon sequencing [12]. The relative risk of having *H. suis* in PD, compared with that in general gastroenterology patients, was 10 (95% confidence interval 3, 33) times greater than that of having *H. pylori* [12]. The frequency of PCR-positivity was greater following *H. pylori* eradication, interpreted as NHPH filling the niche left by eradicating a co-infection. Follow-up showed the hazard-of-death (age at diagnosis- and gender-adjusted) to be 12 times greater with *H. suis* PCR-positivity than with negativity [10]. This is in all-cause mortality. All-cause mortality is not increased with *H. pylori* infection in the general population [13], but there are no corresponding data for NHPH or for *Helicobacter* in PD. No deaths were attributed to peptic ulceration or gastric cancer, as a signature of *Helicobacter* causality [13]. Although chronic gastritis caused by NHPH appears less active and less severe, the risk of developing mucosa-associated lymphoid tissue (MALT) lymphoma is well recognised.

In honing the hypothesis that members of the *Helicobacter* genus, other than *H. pylori*, have a causal role in PD, we (i) examine whether mortality with PD or a pre-presentation manifestation, depression, might be associated with occupational exposure to NHPH; and (ii) confirm whether positivity with the *ureA* PCR [12] does, indeed, exclude *H. pylori.* Within NHPHs, we further investigate the clinical specificity of the *ureA*-based PCR for *H. suis* [14].

## 2. Results

### 2.1. Literature Review

#### 2.1.1. Mortality with PD in Those Occupationally at Risk of NHPH

Table 1 summarises the three epidemiological studies (all in the U.S.) of relative mortality associated with PD which met the inclusion criteria.

Lee et al. (2002) found increased deaths from PD and from non-Hodgkin lymphoma amongst livestock farmers, compared with all decedents [15]. In contrast, proportional mortality from PD was decreased in arable farmers, that from non-Hodgkin’s lymphoma being unaffected. In the six million decedents studied in 26 of the 50 states over a decade, farmers were predominantly white male farmers (42,857 farming livestock, 191,308 crops). PD-specific mortality was 19% higher in these white male livestock farmers compared with non-farmers but 14% lower in crop farmers (proportionate mortality ratios 119 (95% CI (105, 134) and 86 (81, 92), respectively, as compared with that for PD-specific cause amongst all decedents in the database). There was no information as to whether PD and non-Hodgkin lymphoma were comorbidities.

Park et al. (2005) [16]. studied farmers and veterinarians in 22 states over 6 years compared with all decedents, there being a one-year overlap with the above study. They found that, of those under 65 years, the PD-specific mortality odds ratio was excessively high (2.23 (1.47, 3.26) in livestock farmers; less so in all farmers and those in farming occupations with probable pesticide exposure (1.58 (1.08, 2.24) and 1.59 (1.09, 2.26), respectively)). Being a veterinarian was not accompanied by an overall increased risk of mortality with PD. However, stratification into livestock and companion animal practices was not made. 

The third study, by Johnson et al. (2007), found that the PD-specific ratio in abattoir workers (with cattle, pigs and sheep) was 2.5 (1.0, 6.5) when compared with the US population for single state over 4 decades [17]. However, in absolute terms, the excess was small, and any familial relationships within these abattoir workers were unexplored. The corresponding ratio for meat processors was only marginally elevated. 

#### 2.1.2. Depression or Suicide in those Occupationally at Risk of NHPH

The overall emphasis in the seven studies meeting the inclusion criteria [18,19,20,21,22,23,24] was not on quantifying the risk but on investigating the factors associated with depression, suicidal ideation, suicide attempt or suicide itself (Table 2). Santos et al. (2021) quantified the relative risk of suicide in farmers as 1.48 but stressed marked variation according to geographic area [21]. This is similar to the elevated risk (1.6) for Group 6 in the International Standard Classification of Occupations, including skilled forestry and fishery workers as well as farmers and agricultural workers [20]. Platt et al. (2010) ranked proportional mortality from suicide as greater in veterinarians compared with the general population in over half the studies reviewed, significantly so in over a third, but without reference to size of effect [18].

In farmers, impaired mental health had a substantial (36%) association with suicide risk [21]. However, evidence of depression was often covert until near the time of suicide [23]. In veterinarians, one review [22] found that the risk of burnout, anxiety and depressive disorders was increased, but in another [19], there was little evidence for poor mental health except in a young females. In slaughterhouse workers [24], there was a higher prevalence of depression than in all comparator groups in 13 out of 14 studies. In the outlier, the prevalence of depression in butchers equalled that in slaughterhouse workers. 

### 2.2. Evaluation of Probe-Based ureA RT-PCR for H. suis

#### 2.2.1. Inclusivity and Exclusivity

Table 3 confirms that the primers successfully amplified all 24 of the *H. suis* targets, irrespective of source, i.e., whether pig-, macaque- or human-derived. The probe picked up all *H. suis* targets apart from one macaque-derived strain. However, in this case, gel electrophoresis confirmed the amplification, with an amplicon size of around 150 bp, as expected for an *H. suis* target. Cycle threshold (Ct) values produced from the *ureA* assay were consistently good, being mostly below 17 for animal-derived isolates and between 20 and 25 for human-derived isolates. 

Testing exclusivity was divided into two parts: Table 4 gives the analysis of 39 extracts from 21 non-target NHPH species and 2 animal-derived *H. pylori*, and Table 5 gives that of 22 extracts from 21 species of other bacteria closely related to *Helicobacter*. Of the 21 non-target NHPH species, six (*H. bizzozeronii*, *H. cetorum*, *H. cholecystus*, *H. cynogastricus*, *H. felis* and *H. heilmannii*) were detected by the probe, an additional four (*H. ailurogastricus*, *H. baculiformis*, *H. canadenis* and *H. salomonis*) being amplified by the *ureA* primers. None in the non-*Helicobacter* panel were detected by the probe, but cross-reactivity was observed for *Wolinella* and *Salmonella typhimurium* on gel electrophoresis.

Overall, the *ureA* probe-based PCR had an inclusivity of 95.8% and an exclusivity of 59.0%.

#### 2.2.2. Limit of Detection

**Figure 1:**
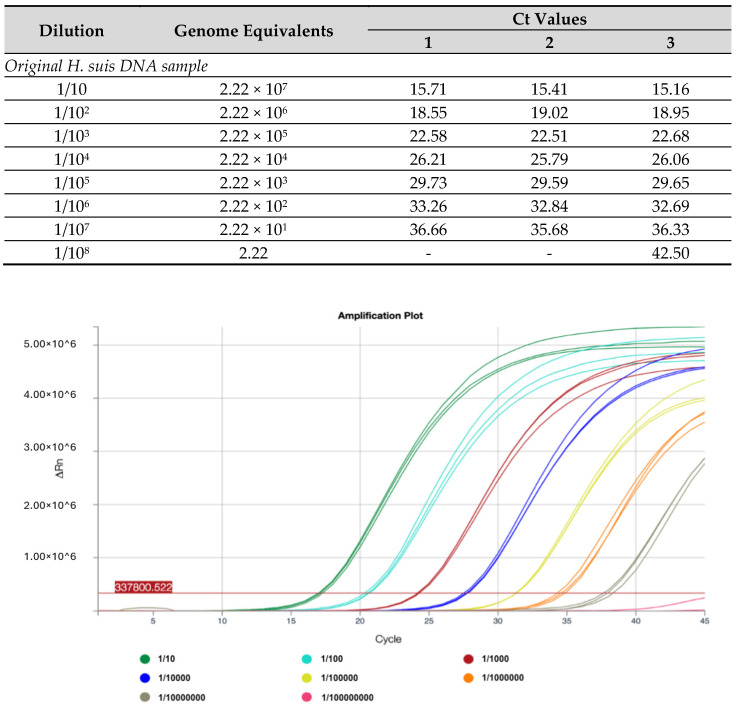
Limit of detection experiment for the ureA assay, tested against a tenfold dilution series of H. suis DNA. Cycle threshold (Ct) values are tabulated (**above**). Amplification plots of a 10-fold dilution series on H. suis DNA samples, containing 2.22 × 10^7^ to 2.22 × 10^1^ bacterial genome equivalents per reaction, are shown (**below**). The baseline threshold (horizontal red line) is set at 337800 to optimise signal-to-noise.

Based on the measured concentration of 40.7 µg/µL, the selected *H. suis* DNA extract would have contained 2.22 × 10^8^ bacterial genome equivalents per (10 µL) reaction. The LoD of the *ureA* assay was tested in triplicate on a 10-fold dilution series, from 1/10–1/10^7^, thus covering a range of 2.22 × 10^7^ to 2.22 × 10^1^ bacterial genome equivalents per reaction. The lowest concentration at which all three replicates produced a Ct value was 1/10^7^. The LoD for the sample was 22.2 genome equivalents with a mean Ct value of 36. Figure 1 shows the amplification plots for the log_10_ dilution series of the original samples. As expected, the greater the variability in Ct values, the lower was the number of target copies available. 

#### 2.2.3. Genome Sequencing and In Silico BLAST Analysis

Sequencing was performed on a random selection of the positive samples amplified by the *ureA* primers to assess the set 95% cut-off point for target identification. Amplicons from pig- and human-derived *H. suis* extracts showed 95–100% homology to *H. suis, Candidatus H. suis* and *Candidatus H. heilmannii*, but macaque-derived *H. suis* strains showed only 85–90% similarity to the *H. suis* reference sequence (Supplementary Table S1). For the four non-target NHPH species tested (*H. bizzozeronii*, *H. felis*, *H. heilmannii* and *H. salomonis*), amplicon sequencing was possible for three, none of which showed a similarity above 95% to *H. suis* reference gene (Supplementary Table S2). 

## 3. Discussion

Livestock farming was clearly associated with more deaths with PD on the death certificate in two national USA studies [15,16], the mortality odds ratio for PD being more than doubled in those under 65 years old in one study [20]. Being a crop farmer was associated with fewer deaths with PD. There was no evidence for more deaths with PD in veterinarians [16] and little evidence for meat processing workers [17]. However, further analysis of the latter two occupations, according to type and intensity of exposure to animals and meat products, is needed. Being an abattoir worker, dealing with cattle, pigs and/or sheep, may double the risk of PD, but only a cohort was compared with population data [17]. Close and high-intensity contact with animals and their products (e.g., saliva, vomitus, faeces, meat or carcass) is a major risk factor for NHPH infections [25]. Thus, hygiene practices are likely to be important determinants of risk, veterinarians being more likely to follow guidelines thoroughly and consistently.

Regarding possible mechanisms and explanations, although proportionate mortality from malignant neoplasms was less overall in farmers, Lee et al. [15]. found that white male livestock farmers, unlike crop farmers, also had greater-than-expected mortality from non-Hodgkin lymphoma (17%) and acute lymphoid (63%) and myeloid (19%) leukaemias. It is well recognised that both *H. pylori* and NHPH are associated with gastric MALT (a non-Hodgkin) lymphoma [26,27]. Further characterisation of the lymphoma associated with livestock farming is needed: does it indeed include MALT (i.e., a primary gastrointestinal, low-grade type B non-Hodgkin) lymphoma, which may regress permanently with the eradication of the *Helicobacter*-related antigenic stimulus? Type B constitutes the majority of non-Hodgkin lymphoma, but MALT lymphoma can progress to the diffuse large B cell type. Risk of death from non-Hodgkin lymphoma (or leukaemia) was the highest in the top-ranking cattle- and pig-farming USA states [28]. Incident cases of non-Hodgkin lymphoma in Canadian male farm residents [29] were increased in those exposed to pigs (when ≥ 13 head) but not to cattle (total 1,262 cases studied), whereas in Finnish farmers [30], there was no relationship to either pig or cattle exposure (750 studied).

The need to explore surrogacy is illustrated in a meta-analysis: [31] the summary risk ratio for the explanation of PD prevalence by ‘pesticide exposure’ (irrespective of type, method of assessment or occupation) varied between 1.34 and 1.56, there being a similar risk ratio for the explanation of non-Hodgkin lymphoma. Moreover, using the blanket term of farming to describe occupation and agrochemical usage is likely to have obscured causal factors. A meta-analysis of 173 studies gave odds ratios of 1.77 (1.48, 2.12) for the association of PD with pesticide usage and 1.26 (1.10, 1.48) for the association with ‘farming/agriculture’ [32]. Were livestock farming split from arable farming [15], the focus may be shifted to zoonosis and narrowed to pesticide usage on livestock (insecticide or fungicide, not herbicide). Interestingly, the same analysis also showed an association (1.21 (1.04, 1.40)) of PD with well water usage: well water might be contaminated by run-off from farms. However, water piped from the municipal reservoir seemed to be an important source of *Helicobacter* infection in less-affluent children in Lima, presumably as a result of faecal contamination *en route* [33].

Since PD is more commonly diagnosed post-retirement, it may be useful to consider pre-presentation manifestations, such as depression, in exploring causality. Farmers are at increased risk of suicide, a scoping review showing mental distress and depression to be a substantial contributory factor [25]. In veterinarians, reviews on mental health were conflicting [18,19,22]. In contrast, abattoir workers had a higher prevalence of depression [24]. However, when considering occupation as a risk factor in mental health and for suicide, comparisons should be made of occupations within the same skill level, since lower-skilled occupations are at greatest risk of suicide [20].

National sources of data, such as death certificates, will, of course, not include potentially important covariates such as farmers’ usage of agrochemicals. Moreover, the occupation of the proband does not encompass that of his family or social circle, and the epidemiology of what originated by zoonosis may now depend on transmission between humans. Our scoping review on the outcomes of depression and suicide mainly retrieved papers describing the contribution of occupational characteristics (such as, in veterinarians, performing euthanasia and access to means of suicide) rather than on quantification of the mental health problems and suicide rate. Regarding the knowledge gap on relative rates of depression in occupations with animal or animal-product exposure, there is a huge resource of original papers to screen (Materials and Methods) but substantial obstacles to address, such as multiple diagnostic methods and ratings. Even simple standardisation (absent, mild, moderate, and severe) of the quantal depression ratings across occupations would have helped. Continuous objective measures of cognition might be ideal in capturing, across countries, some occupational mental health issues. Increased variance in cognitive processing time has been demonstrated in PD [34,35] and might be a pre-presentational manifestation. However, in diagnosed PD, ‘bradyphrenia’ cannot be described simply as a nosological entity, because of the contribution of iatrogenic effects [36].

Non-*Helicobacter pylori Helicobacters,* as a target driver of PD, present a challenge in keeping with the elusiveness of the link between livestock farming and PD. A similar conundrum is being addressed for gastric MALT lymphoma. There, indirect evidence of responsiveness to antimicrobial treatment has been taken on board: 10% of patients with gastric MALT lymphoma are *H. pylori-*negative, but 29% of these show complete histopathological remission after *H. pylori* eradication therapy [37]. Moreover, splitting the *H. pylori*-negative MALT lymphomas by NHPH status showed that anti-*Helicobacter* treatment resulted in complete remission in 75% of the NHPH-positive but only 23% of the NHPH-negative. Indeed, in 2020, the European Society for Medical Oncology Guidelines Committee revised recommendations, from prompt initiation of targeted anti-lymphoma treatment in *H. pylori-*negative early-stage MALT lymphoma, to include a trial of anti-*Helicobacter* therapy [38]. Whether the use of standard anti-*H. pylori* regimens is an adequate approach to NHPH infection is undetermined. Knowledge of antimicrobial sensitivity in humans has been limited by the difficulty in culturing these fastidious bacteria, but resistance patterns of NHPH derived from their animal hosts may be a useful starter.

How big is the problem of NHPH in humans? The true prevalence of gastric NHPH colonisation in humans may be much higher than the literature indicates. In 2020, the genus *Helicobacter* comprised 53 species with validly published names [39]. There are many primary hosts. *Helicobacter pylori* is the most prevalent in humans, its causal role being recognised in chronic gastritis, peptic ulcer and gastric cancer. Emerging evidence points to a role of NHPH in these diseases [40]. In Karachi, in 2012, the prevalence in dyspepsia of the canine and feline NHPHs, *H. heilmannii* and *H. felis*, was reported as 6 and 4%, respectively, all being co-infections with *H pylori* [41]. In Japan, in 2017, the prevalence of NHPH in patients undergoing gastroscopy was reported as 6% [42], but, by 2020, as 21%, with the porcine NHPH, *H. suis*, being the most prevalent species [43]. In Belgium, in 2023, NHPH prevalence was reported as 28–29% of those undergoing gastroscopy, the canine and feline NHPH, *H. bizzozeronii* and *H. felis*, being the most common [44]. It was ventured that pets, colonised by several different species, may act as reservoirs. In the case of *H. suis*, raw or undercooked pork meat, as well as contact with pigs, may result directly in zoonosis [45]. The NHPH problem created by species colonising the lower intestinal tract of animals, many of which can also colonise the human guts, is relatively unexplored. These *Helicobacters* are often associated with diarrhoea and can cause bacteraemia and systemic disease. Their relevance to PD, in which colitis, systemic disease (such as hot sweats) and skin involvement (rashes, cellulitis) are features, is, to say the least, engaging [46].

How easy is NHPH to detect? The answer is dependent on the tools applied. The current gold standard for detection is molecular microbiology on endoscopic biopsy combined with sequencing. Methods used for *H. pylori* detection have low sensitivity to NHPH: rapid urease tests on gastric biopsies (40% pick-up if present), urea breath tests (15%), antibody detection in blood (23%), immunohistochemistry (40%), and stool antigen tests (0%) [47]. Moreover, a retrospective review of the detection of gastric NHPH by clinical histopathologists showed that 20% of cases were missed [44]. The sparsity of NHPH compared with *H. pylori* and the focal (e.g., with one gastric crypt loaded but no NHPH in the next section^11^) and patchy antral distribution make detection difficult. (Low density of NHPH colonisation in humans is generally noted compared with that in the natural host.) Screening saliva to predict the presence of gastric NHPH may be useful. In mouth swabs from patients with chronic gastritis, peptic ulcer disease or gastric MALT lymphoma, the canine and feline NHPH species were most frequently detected, with *H. felis* at 10% and *H. bizzozeronii* at 5%, followed by *H. suis* and *H. salomonis*, each at 2% [44]. Co-infections were noted with other NHPH and with *H. pylori*. The stability of salivary NHPH content and its relationship to gastric colonisation need further definition. It may well be that matrix-assisted laser desorption/ionisation mass spectrometry (MALDI-TOF MS) will, in the future, add timely information on species identification directly from clinical samples and on antibiotic resistance.

The limitation in studying those occupationally at risk of NHPH zoonosis and those diseases associated with NHPH, probably–causally (MALT lymphoma) or circumstantially (PD), lies in the detection of NHPH infection. Multiple NHPH are already known to infect humans, including *H. bilis, H. bizzozeronii, Candidatus H. bovis, H. canadensis, H. canis, H. cinaedi, H. felis, H. fennelliae, H. ganmani, H. heilmannii, H. hepaticus, H. pullorum, H. salomonis, H. suis* and *H. winghamensis.* The frequency of co-infection with *H. pylori* is unknown. A broad-based, rapid, non-invasive approach to screening for *Helicobacter* genus is needed, followed by biopsy-based attribution to species.

Regarding the evaluation of *ureA* PCRs for *H. suis* detection, as expected, the primers were indiscriminate, amplifying 10 other NHPH species, in keeping with the occasional false positivity previously reported [14]. However, the specificity for *H. suis* detection becomes acceptable when complemented by amplicon sequencing and a 95% cut-off for sequence similarity. Confirming *H. suis* infection is challenging due to unconfirmed (Candidatus) and uncultured (unidentified) *Helicobacter* species and possible dissimilarities in *H. suis* according to host (as seen here when comparing a macaque source). Our optimised *ureA* probe-based PCR did not meet acceptable exclusivity criteria for a *H. suis*-specific monoplex PCR, since multiple non-target species were amplified and detected. Nonetheless, the sensitivity was very high in detecting all strains of *H. suis* tested, including the macaque-derived strains missed by the original PCR.

Importantly, neither *ureA* PCR assay amplified *H. pylori* in vitro. That is, the relative risk of having gastric NHPH, but not necessarily *H. suis*, was indeed 10 times greater than that of having *H. pylori* in people with PD compared with gastroenterology patients [12].

## 4. Materials and Methods

### 4.1. Literature Reviews in Those Occupationally Exposed to Animals or Their Products

#### 4.1.1. Is There Increased Mortality with PD?

**Figure 2:**
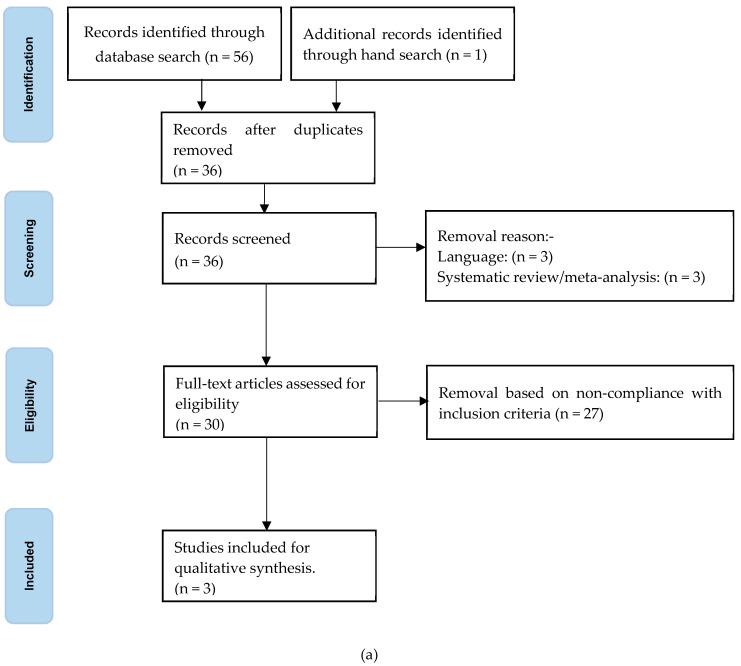

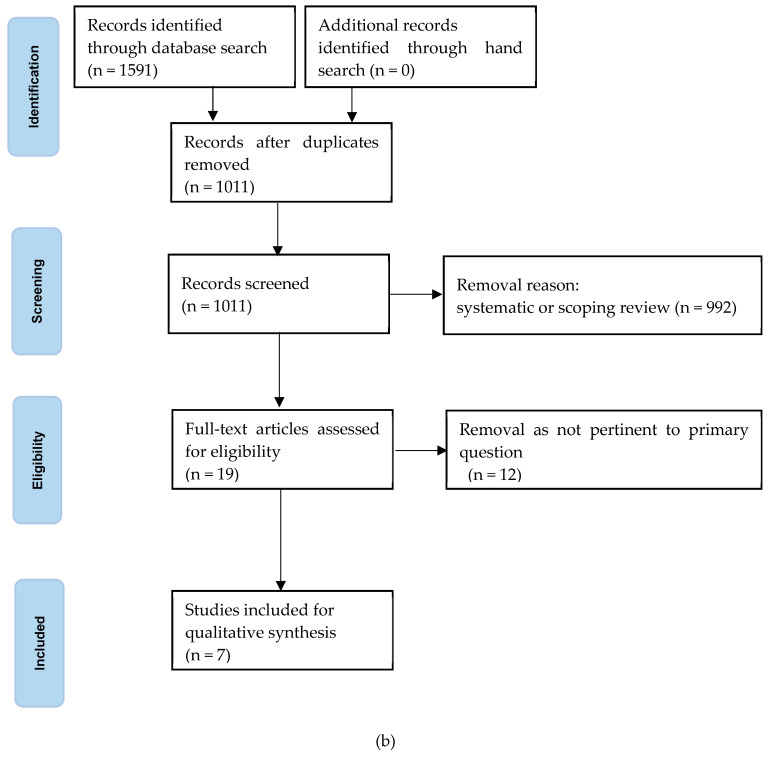
Flow chart based on PRISMA guidelines for (**a**) systematic review of mortality associated with idiopathic Parkinsonism in occupations with exposure to animals and/or their products, and (**b**) scoping review of depression and suicide in occupations with exposure to animals and/or their products.

The systematic search combined three groups of keywords: relating to Parkinson’s disease, occupation with exposure to animals or their products (farmer, meat processer, abattoir worker, butcher or veterinarian) and mortality rate. Figure 2a gives the stages of the systematic review (identification, screening, eligibility assessment and the inclusion decision) in line with the “Preferred Reporting Items for Systematic Reviews and Meta-Analyses” (PRISMA) guidelines [48]. The Ovid Medline, Embase, Global Health and PsycINFO databases were used to search for articles published in peer-reviewed scientific journals between 1946 and 2023. The screening of sources, titles and nature of publication (by reviewer W.W.) excluded those not primarily in English or without a translation into English; case histories; book chapters/conference proceedings/letters/comments/reviews; systematic review or meta-analysis; and those focussing on occupational exposure to toxins or hazards such as solvents, metals, pesticides/herbicides or magnetic fields. Secondary reviewers (R.J.D. and S.M.D.) assessed any equivocal selections. Studies obeying the inclusion criteria were eligible: large-cohort/national-level studies of PD as a cause of death, with clearly defined occupations. The following information was extracted from each included article: (i) citation; (ii) geographical region; (iii) source of mortality ascertainment; (iv) cohort size; and (v) outcome findings.

#### 4.1.2. Is There Increased Depression or Suicide?

Of the three key word groupings in Section 4.1.1, occupation with exposure to animals or their products was retained, depression or suicide was substituted for mortality rate, and Parkinson’s disease was removed. We homed in on depression, since it is a manifestation of PD which is likely to be documented irrespective of clinical diagnosis and might flag the PD prodrome. At 5 years before diagnosis, participants who went on to develop PD had a higher risk ratio for depression [49]. The search in Figure 2b identified 1011 original articles (364 published in last five years), 19 of which were published as systematic or scoping reviews or meta-analyses and went forward to eligibility checking. A scoping review of eligible articles was used to clarify key concepts, home in on answers to research questions and identify gaps in the knowledge base [50].

### 4.2. Validation and Optimisation of the ureA PCR

#### 4.2.1. Original *ureA*-Based PCR for *H. Suis*

This quantitative SYBR green real-time (RT)-PCR, based on the *ureA* gene, was intended to be species-specific for *H. suis* detection in gastric biopsies, with positive results subject to confirmation by amplicon sequencing [12]. Urease permeates through *Helicobacter* species: sequencing of the positive PCR amplicons was essential for identification of *H. suis* and any false positives corresponding to the *ureA* gene sequence of non-target *Helicobacters*. For generating the standard, part of the *ureA/B* gene cluster (1236 bp) from an archived *H. suis* strain was amplified using primers U430F and U1735R [51]. The sense primer was BF_HsuisF1: 5′-AAA ACA MAG GCG ATC GCC CTG TA-3′, anti-sense primer BF_HsuisR1: 5′-TTT CTT CGC CAG GTT CAA AGC G-3′, annealing temperature 62 °C. Standards and samples were run in duplicate on a CFX96^TM^ RT-PCR System with a C1000 Thermal Cycler (Bio-Rad, Hercules, CA, USA). All DNA extracts were assayed on two separate occasions.

The suitability of the primers was assessed in silico. Primers were aligned against sequences of 12 strains of *H. suis* (HS1, HS2, HS3, HS-HA, SH4, SH8, SH10, SNTW101c, NHP19-4022, NHP19-4004, NHP19-4003 and NHP19-0020) obtained from the National Center for Biotechnology Information (NCBI) database: there was a perfect match to every strain using the multiple sequence alignment program, Clustal Omega (https://www.ebi.ac.uk/jdispatcher/msa/clustalo, accessed on 1 July 2024) [52]. The amplicons of the amplified *H. suis* region (150 bp) were then entered into the Basic Local Alignment Search Tool (BLAST) (https://blast.ncbi.nlm.nih.gov/Blast.cgi, accessed on 1 July 2024) to confirm the specificity for the *H. suis* target. When the maximum number of targets of the search was set to 500, apart from *H. suis* strains, there was sequence similarity, above the 95% cut-off [12], to a number of ‘Candidatus *H. heilmannii*’ strains submitted in 2016, but to no other species. It is reasonable to suspect that the Candidatus *H. heilmannii* was, in fact, unidentified *H. suis*. The short amplicon of 150 bp could have impaired ‘read quality’ during sequencing, with difficulty in subsequent speciation via the BLAST platform. Speciation using sequences from short fragments will become more difficult as the database incorporates more DNA sequences from newly described strains and species.

#### 4.2.2. Redesign as a Probe-Based *ureA* PCR

To improve specificity and detection accuracy, the original assay was redesigned into a probe-based PCR with no modification of the primers. Sequences of the *Helicobacter ureA* gene were searched for in the NCBI database: sequences of the 21 species obtained were aligned against the amplified region using Clustal Omega. Of these, sequences of five species were distinctively different from the rest (*H. pylori*, *H. jaachi*, *H. trogontum*, *H. mehlei* and *H. marmotae*) and excluded from the alignment. The probe was designed based on a region with sufficient sequence variation between the remaining 16 species (Supplementary Figure S1) to allow at least three mismatches between H. suis targets and non-target Helicobacters. Sufficient mismatches should avoid cross-reactivity with non-target strains. The candidate probe was 5′-ACG ACT TGA CAT CGC CTC TGG TAC AGC-3′. It was checked for self-complementarity, guanine and cytosine (GC) content and melting temperature, using an online oligonucleotide property calculator, OligoCalc (http://biotools.nubic.northwestern.edu/OligoCalc.html, accessed on 1 July 2024). There was no self-complementarity, and the GC content was appropriate, the annealing being 71.5 °C.

The probe had a standard concentration of 250 nmol/L. Reactions were prepared to a final volume of 30 µL as follows: 10 µL of DNA template, 15 µL of QuantiTect Multiplex PCR NoRox Buffer, 4 µL of water and 1 µL of primer and probe detection mix (made up of 10 µL forward primer, 10 µL reverse primer, 5 µL probe buffer and 75 µL of Tris-EDTA buffer). Reactions were optimised for use with the Applied Biosystems QuantStudio 7 Flex real-time PCR platform. The optimised cycling conditions were 95 °C for 15 min, 45 cycles of denaturation at 95 °C for 15 s, annealing at 65 °C for 30 s and elongation at 72 °C for 90 s.

#### 4.2.3. Analytical Specificity

The analytical validity of the optimised assay was assessed using target and non-target bacterial panels.

The target *Helicobacter* panel was composed of DNA extracts from 20 *H. suis* isolates from pigs and 2 from macaques (Laboratory of Veterinary Bacteriology and Mycology, Faculty of Veterinary Medicine, Ghent University) and 2 *H. suis* strains isolated from humans (National Institute of Infectious Diseases and Kitasato University, Tokyo, Japan). There were two non-target panels: one of non-target *Helicobacters*, the other of bacterial species closely related to *Helicobacter*.

The non-target *Helicobacter* panel comprised DNA extracts from (i) 2 strains of *H. pylori* (Ghent University), (ii) 18 NHPH species represented by 35 extracts (Ghent University): *H. acinonychis*, *H. ailurogastricus*, *H. bilis*, *H. baculiformis*, *H. bizzozeronii*, *H. canadenis*, *H. cetorum*, *H. cholecystus*, *H. cynogastricus*, *H. equorum*, *H. felis*, *H. heilmannii*, *H. marmotae*, *H. mesocricetorum*, *H. mustellae*, *H. pullorum*, *H. salomonis* and *H. trogontum*, and (iii) 1 additional strain of *H. heilmannii* and 3 additional other *NHPH* species (*H. cinaedi, H. canis* and *H. fennelliae*) (Deutsche Sammlung von Mikroorganismen und Zellkulturen, DSMZ).

The non-*Helicobacter* panel was made up of the following: (i) 20 species available represented by 21 extracts (Ghent University): *Actinobacillus equuli*, *Aeromonas hydrophila*, *Bacillus licheniformis*, *Campylobacter fetus*, *C. jejuni*, *C. upsaliensis*, *Enterococcus faecalis*, *Escherichia coli*, *Fusobacterium gastrosuis*, *Klebsiella pneumoniae*, *Mannheimia haemolytica*, *Pasteurella multocida*, *Proteus vulgaris*, *Pseudomonas aeruginosa*, *Salmonella typhimurium*, *Staphylococcus aureus*, *Streptococcus equi* subsp. *Equi*, *Streptococcus equi* subsp. *Zooepidemicus*, *Streptococcus pseudintermedius*, *Streptococcus suis* and *Yersinia pseudotuberculosis* and (ii) 1 further species (DSMZ): *Wolinella succinogenes*, an epsilon subclass of *Proteobacteria*, which is closely related to *H. pylori*, *H. hepaticus* and *Campylobacter jejuni*.

To confirm inclusivity and exclusivity of the assay, all DNA extracts from the target panel and non-target panels were tested under optimal conditions, with one positive control (PC) and one no-template control (NTC) of water in each run. Gel electrophoresis was run on PCR products to confirm amplicon size and exclude non-target amplifications

#### 4.2.4. Analytical Sensitivity

The limit of detection (LoD) was tested following the optimisation of the PCR programme. To ascertain the concentration of the DNA targets, several samples of the archived *H. suis* DNA extract were examined using a Nanodrop spectrophotometer. The most adequate one had a 260/280 ratio of 1.80 and a concentration of 40.7 µg/µL; it was considered to be a ‘pure’ sample and was selected for LoD testing.

A tenfold (1/10–1/10^8^) dilution set was prepared and assayed, in triplicate, with *H. suis* DNA in Tris-EDTA buffer. The dilution series was tested using the above optimised concentration of each primer and the probe. The assay was run on the Applied Biosystems QuantStudio 7 Flex real-time PCR system under the optimised condition, as follows: 95 °C for 15 min, 45 cycles of the denaturation stage at 95 °C for 15 s, and 65 °C for 30 s.

#### 4.2.5. Amplicon Sequencing and In Silico BLAST Analysis

Sanger sequencing (Animal and Plant Health Agency (APHA), Addlestone, UK) was performed on randomly selected, PCR-positive *H. suis* samples and non-target NHPH samples to confirm speciation and detect any false positives corresponding to the *ureA* gene sequence of non-target *Helicobacter* species.

Read quality was assessed, and low-quality segments were removed. Sequences generated by forward and reverse primers were aligned using Clustal Omega, the consensus double-stranded area between the two sequence reads being cropped and entered into BLAST for comparison with the *H. suis* reference sequence (Assession Number NZ_AP023046) for similarities. Similarity to the reference, as well as to the top 5% of the matched *Helicobacter* species, was reported.

## 5. Conclusions

**Figure 3:**
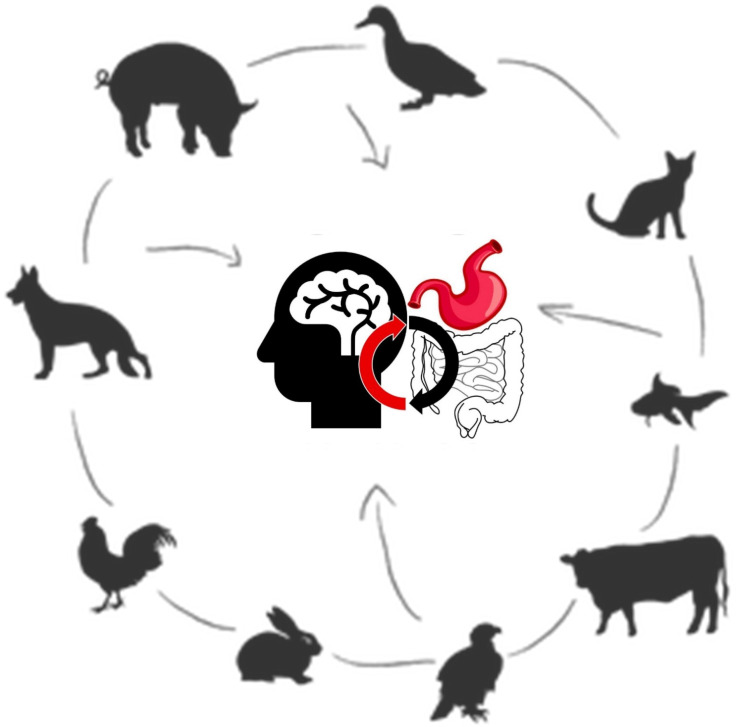
Summary of the major findings. From the literature review: (i) the association of Parkinson’s disease (PD) deaths with livestock farming; (ii) the excess non-Hodgkin lymphoma in livestock farmers compatible with non-*Helicobacter pylori Helicobacter* (NHPH) causality; and (iii) the link between gastric NHPH and PD described using PCR, targeting porcine *Helicobacter*. From the current investigation: the redesign into a probe-based PCR confirmed *H. pylori* exclusion but showed a lack of specificity within NHPH. Further exploration of the zoonotic hypothesis requires a pan-*Helicobacter* screen.

Figure 3 summaries the major findings.

There is considerable indicative and circumstantial evidence that zoonosis may be the cause of excess deaths with PD in livestock farmers. A candidate causal agent is the genus *Helicobacter*: a high prevalence of *H. pylori* has been associated with PD, and the disease-modifying effect of its eradication has been demonstrated [1]. It is now important to determine the relative prevalence of NHPH in occupations at particular risk of PD. Proof of causality will rest with whether proven NHPH eradication in PD is disease-modifying, a more difficult study to design than the placebo-controlled, biopsy-proven, *H. pylori* eradication study [6]. Non-invasive screening of saliva and stools is required for the detection of gastric, intestinal or enterohepatic NHPH. Unlike *H. pylori*, gastric NHPH load is usually too low in humans for the urea breath test to be a definitive screen [12], and sparsity in the gastric mucosa means multiple biopsies are likely to be needed for a pan-*Helicobacter* PCR and amplicon sequencing. From an occupational health perspective, the screen needs to be readily available for those at risk. Defining which particular species of *Helicobacter* are implicated would help focus resources.

Different groups of *Helicobacter* species have been classified as gastric, enteric and enterohepatic, have different animal hosts and have different associated pathology. It is thus counterintuitive that the species cannot be better grouped by molecular microbiology: PCR assays developed for subtyping might be added to a pan-*Helicobacter* screen. This would be clinically useful in planning diagnostic biopsy and might predict antimicrobial sensitivity. Concurrent presence of more than one species must be anticipated. More rational and clinically useful taxonomic classification of *Helicobacters* may be possible.

## Figures and Tables

**Table 1:** Large scale or national level studies of mortality with PD in occupations with exposure to animals or their products.

Reference	Country/Cohort (Sample Size)	Occupations	Source of Mortality Ascertainment	Findings
Lee et al., 2002 [15]	26 U.S. states, 1984–1993. (6 million deaths)	White male livestock farmers (40 thousand deaths); crop farmers (190 thousand).	Occupation and industry-coded U.S. death certificate data.	White male livestock farmers, had higher mortality with PD than non-farmers, white male crop farmers had lower.
Park et al., 2005 [16]	22 U.S. states, 1992–1998. (2.6 million deaths)	All farmers (150 thousand deaths); livestock farmers (120 thousand); veterinarians (not stated).	Death certificate data from National Occupational Mortality Surveillance System.	Below 65 years, marked excess deaths with PD in livestock farmers, but less so when considering all farmers. No excess with PD in veterinarians.
Johnson et al., 2007 [17]	Baltimore, Maryland, Meat cutters union, 1949–1989. (2392 deaths in meat workers)	Abattoir (1380 deaths); meat processing (1012).	Union records; Social security mortality files; Maryland Departments of Motor Vehicle and Vital Records; National Death Index; Pension Benefit Information Company; personal letter or telephone call.	Excess of deaths with PD in abattoir and, to a lesser extent, in meat processing plant workers compared with U.S. general population.

**Table 2:** Reviews and meta-analysis comparing depression and suicide by occupational exposure to animals or their products.

Reference	Number of Studies	Occupation	Outcome	Findings
Platt et al., 2010 [18]	19 studies in systematic review (1806–2008)	Veterinarians	Suicide	11/19 studies found proportional mortality ratio in veterinarians numerically increased compared with general population; increases were significant (*p* < 0.05) in 7 studies.
Platt et al., 2012 [19]	52 studies in systematic review (to 2008)	Veterinarians	Non-fatal suicidal behaviour, mental health difficulties, stress and burnout, occupational difficulties, psychological characteristics	Little evidence that veterinarians have particularly poor mental health. However, young and female veterinarians appeared at higher risk of suicidal thoughts and mental health difficulties.
Milner et al., 2013 [20]	34 in meta-analysis (1950 to 2012)	International Standard Classification of Occupations (ISCO) 2008)	Suicide	Significantly elevated risk rate ratio (95% CI) of 1.6 (1.2,2.3) in ISCO Group 6, including farmers and agricultural workers, but not 0.7 (0.5, 0.9) in Group 1 including professionals, compared with the working-age population. Lowest skilled occupations at greater risk: Group 8, elementary professions ratio 1.84 (1.46–2.33); Group 9, machine operators and deck crew 1.78 (1.22–2.60).
Santos et al., 2021 [21]	32 in meta-analysis, 14 in systematic review (performed 2021)	Farmers	Suicidal ideation, suicide attempt or suicide	Meta-analysis quantified excess risk of suicide in farmers at 1.48, varying according to geographical area. Systematic review in which there was proportional contribution of associated factors: 35.7% attributed to mental health, including feeling of self-blame/stress. Suicide ideation associated with mental distress/ depression.
Pohl et al., 2022 [22]	21 studies in scoping review (2000 to 2021)	Veterinarians	Mental wellbeing	Risks of burnout, anxiety and depressive disorders higher than in general population.
Purc-Stephenson et al., 2023 [23]	14 studies in systematic review (1980–2023)	Farmers	Mental health stigma	Many either diagnosed with or showed symptoms of depression, anxiety or distress at time of death, but had hidden their problem.
Slade et al., 2023 [24]	14 studies in systematic review (to 2018)	Slaughterhouse workers	Mental health disorders	Significantly higher prevalence rate of both depression and anxiety in 13/14 studies than in comparator group.

**Table 3:** Inclusivity of the *ureA* probe-based PCR for *H. suis* using the target panel.

Class: Epsilonproteobacteria				
Order: Campylobacterales				
Family: Helicobacteraceae				
Species	Isolates	Amplified by *ureA* Primers	Detected by *ureA* Probes	Ct Value
***H. suis*** ***(pig-derived)***	HS1	Yes	Yes	12.42
	HS2	Yes	Yes	14.43
	HS3	Yes	Yes	12.87
	HS4	Yes	Yes	13.87
	HS5	Yes	Yes	14.73
	HS6	Yes	Yes	10.97
	HS7	Yes	Yes	13.51
	HS8	Yes	Yes	11.81
	HS9	Yes	Yes	12.58
	HS10	Yes	Yes	14.36
	P13/04	Yes	Yes	12.53
	P13/24	Yes	Yes	16.09
	P13/26	Yes	Yes	11.91
	P13/28	Yes	Yes	14.40
	P13/32	Yes	Yes	14.27
	P13/35	Yes	Yes	12.26
	P13/36	Yes	Yes	14.41
	P14/06	Yes	Yes	16.81
	P14/09	Yes	Yes	15.52
	P14/10	Yes	Yes	15.56
***H. suis*** ***(macaque-derived)***	HSMf331	Yes	No	-
	HSMF504/1	Yes	Yes	13.89
***H. suis*** ***(human-derived)***	NHP19-4004	Yes	Yes	21.03
	NHP19-4022	Yes	Yes	25.17

**Table 4:** Exclusivity of the *ureA* probe-based PCR for *H. suis* using the non-target *Helicobacter* panel.

Class: Epsilonproteobacteria				
Order: Campylobacterales				
Family: Helicobacteraceae				
Species	Isolates	Amplified by *ureA* Primers *	Detected by *ureA* Probes *	Ct Value
***H. acinonychis***	H.acino 1	No	No	-
	H.acino 2	No	No	-
	H.acino 3	No	No	-
***H. ailurogastricus***	ABS7.1	Yes	No	-
	ABS 9.4	Yes	No	-
	ASB 11	Yes	No	-
	ASB 21	Yes	No	-
***H. bilis***	ATCC 49320	No	No	-
***H. baculiformis***	M50	Yes	No	-
***H. bizzozeronii***	R53	Yes	Yes	33.45
	t10	Yes	Yes	32.16
	ASB22	Yes	Yes	30.66
	Yryla	Yes	Yes	30.22
	Heydar	Yes	Yes	34.66
***H. canadenis***	H7163	Yes	No	-
***H. canis***	CCUG 32756	No	No	-
***H. cetorum***	MIT 015903	Yes	Yes	40.48
	MIT 016202	Yes	Yes	37.88
***H. cholecystus***	R3555	Yes	Yes	30.49
***H. cinaedi***	DSM 5359	No	No	-
***H. cynogastricus***	JMK4	Yes	Yes	16.16
***H. equorum***	EqF1	No	No	-
***H. felis***	DS1	Yes	Yes	17.66
	M26	Yes	Yes	20.97
	M29	Yes	Yes	23.89
	M38	Yes	Yes	40.80
	M39	Yes	Yes	36.44
***H. fennelliae***	DSM 7491	No	No	-
***H. heilmannii***	ABS 1.4	Yes	Yes	20.06
	ABS 1	Yes	Yes	20.60
***H. marmotae***	CB55	No	No	-
***H. mesocricetorum***	CCUG 45420	No	No	-
***H. mustellae***	LMG 8776	No	No	-
***H. pullorum***	CB103	No	No	-
***H. pylori***	Qi3	No	No	-
	STM0138	No	No	-
***H. salomonis***	M45	Yes	No	-
	Elvira II	Yes	No	-
	Alman0595	Yes	No	-
	Innenen	Yes	No	-
***H. trogontum***	R3554	No	No	-

* amplified by primer = 26/41 (63%); detected by probe = 16/41 (39%).

**Table 5:** Exclusivity of *ureA* probe-based PCR for *H. suis* using the non-*Helicobacter* panel.

Class	Order	Family	Species	Isolates	Amplified by *ureA* Primers *	Detected by *ureA* Probes *
Epsilonproteo-bacteria	Campylobacterales	*Helicobacteraceae*	*Wolinella succinogenes*	DSM1740	Cross-reactivity	No
		*Campylobacteraceae*	*C. fetus*	V24-6	No	No
			*C. jejuni*	KC40	No	No
				TB4	No	No
			*C. upsaliensis*	LMG19529	No	No
Gammaproteobacteria	Aeromonadales	*Aeromonadaceae*	*Aeromonas* *hydrophila*	LR3758	No	No
	Enterobacteriales	*Enterobacteriaceae*	*Escherichia coli*	LR3889	Yes	No
			*Klebsiella pneumoniae*	LR3806	No	No
			*Proteus vulgaris*	LR1537	No	No
			*Salmonella typhimurium*	LR3862	Cross-reactivity	No
			*Yersinia pseudotuberculosis*	LR3798	No	No
	Pasteurellales	*Pasteurellaceae*	*Actinobacillus equuli*	CCUG 19799T	No	No
			*Mannheimia haemolytica*	LR3870	No	No
	Pseudomonadales	*Pseudomonadaceae*	*Pasteurella multocida*	LR3892	No	No
			*Pseudomonas aeruginosa*	LR3861	No	No
Bacilli	Bacillales	*Bacillaceae*	*Bacillus licheniformis*	DSM347	No	No
		*Staphylococcaceae*	*Staphylococcus aureus*	LR3843	No	No
			*Staphylococcus pseudintermedius*	LR3821	No	No
			*Streptococcus equi* subsp. *Equi*	LR3876	No	No
			*Streptococcus equi* subsp. *Zooepidemicus*	LR3883	No	No
			*Streptococcus suis*	LR3841	No	No
	Lactobacillales	*Enterococcaceae*	*Enterococcus faecalis*	LR 3751	No	No
Fusobacteria	Fusobacteriales	*Fusobacterium*	*Fusobacterium gastrosuis*	CDW01	No	No

* amplified by primer = 1/23 (4%); detected by probe = 0/23 (0%).

## Data Availability

The data are given in this article and Supplementary Materials

## Supplementary Materials

The following supporting information can be downloaded at: https://www.mdpi.com/article/10.3390/ijms252313123/s1.
