# Supplementary material for: Non-Helicobacter pylori Helicobacters, a Treatable Provocateur of Parkinson’s Disease: Hypothesis, Evidence and Species Specificity
Source: Int J Mol Sci. 2024 Dec 6;25(23):13123. doi: 10.3390/ijms252313123 (PMC11642523; doi:10.3390/ijms252313123)
Supplement: Supplementary file 1 [file ijms-25-13123-s001.zip › ijms-3318661-supplementary.pdf]

Non-*Helicobacter pylori* Helicobacters, a treatable provocateur of Parkinson's disease: hypothesis, evidence and species specificity

Supplementary Tables and Figure

Supplementary Table S1. **Amplicon sequencing analysis of the *H. suis* target panel, showing the amplicon length, top 5% Helicobacter species identified and similarity to *H. suis* reference gene.**

H. suis	Strain	Amplicon length (bp)	Top 5% *Helicobacter* species identified	Accession no.	Sequence similarity %
**Pig-derived**	HS6	41	H. suis	AP023046.1	**100**
			Candidatus *H. heilmannii*	AB498750.1	100
			Candidatus *H. suis*	EF204594.1	100
			Candidatus *H. heilmannii*	AF508015.1	97.56
	HS7	64	*H. suis*	AP023046.1	**100**
			Candidatus *H. heilmannii*	AB498750.1	100
			Candidatus *H. suis*	EF204594.1	100
			Candidatus *H. heilmannii*	AF508009.1	98.44
			Candidatus *H. heilmannii*	AF508015.1	95.31
	HS8	64	*H. suis*	AP023046.1	**100**
			Candidatus *H. heilmannii*	AB498750.1	100
			Candidatus *H. suis*	EF204594.1	100
			Candidatus *H. heilmannii*	AF508009.1	98.44
			Candidatus *H. heilmannii*	AF508015.1	95.31
	HS9	64	*H. suis*	AP023046.1	**100**
			Candidatus *H. heilmannii*	AB498750.1	100
			Candidatus *H. suis*	EF204594.1	100
			Candidatus *H. heilmannii*	AF508009.1	98.44
			Candidatus *H. heilmannii*	AF508015.1	95.31
	HS10	63	*H. suis*	AP023046.1	**98.44**
			Candidatus *H. heilmannii*	AB498750.1	98.44
			Candidatus *H. suis*	EF204594.1	98.44
			Candidatus *H. heilmannii*	AF508006.1	98.41
			Candidatus *H. heilmannii*	AF508009.1	96.88
	P13/04	64	*H. suis*	AP023046.1	**100**
			Candidatus *H. heilmannii*	AB498750.1	100
			Candidatus *H. suis*	EF204594.1	100
			Candidatus *H. heilmannii*	AF508009.1	98.44
			Candidatus *H. heilmannii*	AF508015.1	95.31
	P13/24	63	*H. suis*	AP023046.1	**100**
			Candidatus *H. heilmannii*	AB498750.1	100
			Candidatus *H. suis*	EF204594.1	100
			Candidatus *H. heilmannii*	AF508009.1	98.41
			Candidatus *H. heilmannii*	AF508015.1	95.24
	P13/26	63	*H. suis*	AP023046.1	**100**
			Candidatus *H. heilmannii*	AB498750.1	100
			Candidatus *H. suis*	EF204594.1	100
			Candidatus *H. heilmannii*	AF508009.1	98.41
			Candidatus *H. heilmannii*	AF508015.1	95.24
	P13/28	63	*H. suis*	AP023046.1	**100**
			Candidatus *H. heilmannii*	AB498750.1	100

			Candidatus *H. suis*	EF204594.1	100
			Candidatus *H. heilmannii*	AF508009.1	98.41
			Candidatus *H. heilmannii*	AF508015.1	95.24
	P13/32	64	*H. suis*	AP023046.1	**100**
			Candidatus *H. heilmannii*	AB498750.1	100
			Candidatus *H. suis*	EF204594.1	100
			Candidatus *H. heilmannii*	AF508009.1	98.44
			Candidatus *H. heilmannii*	AF508015.1	95.31
	P13/35	64	*H. suis*	AP023046.1	**100**
			Candidatus *H. heilmannii*	AB498750.1	100
			Candidatus *H. suis*	EF204594.1	100
			Candidatus *H. heilmannii*	AF508009.1	98.44
			Candidatus *H. heilmannii*	AF508015.1	95.31
	P13/36	64	*H. suis*	AP023046.1	**100**
			Candidatus *H. heilmannii*	AB498750.1	100
			Candidatus *H. suis*	EF204594.1	100
			Candidatus *H. heilmannii*	AF508009.1	98.44
			Candidatus *H. heilmannii*	AF508015.1	95.31
	P14/06	64	*H. suis*	AP023046.1	**100**
			Candidatus *H. heilmannii*	AB498750.1	100
			Candidatus *H. suis*	EF204594.1	100
			Candidatus *H. heilmannii*	AF508009.1	98.44
			Candidatus *H. heilmannii*	AF508015.1	95.31
	P14/09	64	*H. suis*	AP023046.1	**100**
			Candidatus *H. heilmannii*	AB498750.1	100
			Candidatus *H. suis*	EF204594.1	100
			Candidatus *H. heilmannii*	AF508009.1	98.44
			Candidatus *H. heilmannii*	AF508015.1	95.31
	P14/10	64	*H. suis*	AP023046.1	**100**
			Candidatus *H. heilmannii*	AB498750.1	100
			Candidatus *H. suis*	EF204594.1	100
			Candidatus *H. heilmannii*	AF508009.1	98.44
			Candidatus *H. heilmannii*	AF508015.1	95.31
**Macaque-derived**	HSMf 331	54	*H. suis*	AP023046.1	**90.57**
			Candidatus *H. heilmannii*	AF508007.1	96
				AF508006.1	96
				AB252065.1	96
	HSMf 504/1	54	*H. suis*	AP023046.1	**86.59**
			Candidatus *H. heilmannii*	AB252065.1	96.23
				AF508007.1	96.23
				AF508006.1	96.23
**Human derived**	NHP19-4004	63	*H. suis*	AP023046.1	**100**
			Candidatus *H. heilmannii*	AB498750.1	100
			Candidatus *H. suis*	EF204594.1	100
			Candidatus *H. heilmannii*	AF508009.1	98.41
			Candidatus *H. heilmannii*	AF508015.1	95.24
	NHP19-4022	64	*H. suis*	AP023046.1	**100**
			Candidatus *H. heilmannii*	AB498750.1	100
			Candidatus *H. suis*	EF204594.1	100
			Candidatus *H. heilmannii*	AF508009.1	98.44
			Candidatus *H. heilmannii*	AF508015.1	95.31

Supplementary Table S2. **Amplicon sequencing analysis of the Non-target *Helicobacter* panel, showing the amplicon length, top 5% Helicobacter species identified and similarity to *H. suis* reference gene.**

Sample	Strain	Amplicon length (bp)	Top 5% *Helicobacter* species identified	Sequence similarity %	Similarity to *H. suis %*
H. bizzozeronii	T10	72	***H. bizzozeronii***	**88.57**	**84.72**
H. felis	M29	64	***H. felis***	**98.44**	**86.92**
			uncultured *Helicobacter* sp.	100	
			Candidatus *H. heilmannii*	98.44	
			uncultured *Helicobacter* sp.	96.88	
			uncultured *Helicobacter* sp.	95.38	
H. heilmannii	ABS 1.4	61	***H. heilmannii***	**100**	**88.3**
			uncultured *Helicobacter* sp.	95.08	
	ABS 1	55	***H. heilmannii***	**100**	**90**
H. salomonis	M45	106	uncultured *Helicobacter* sp.	95.37	**87.04**
			H. salomonis	**95.37**	
	Elvira II	48	***H. salomonis***	**95.83**	**72.19**
			uncultured *Helicobacter* sp.	100	
			H. felis	95.83-95.74	
			uncultured *Helicobacter* sp.	95.74	

```
                    Forward primer (BF_HsuisF1)
                    AAAACAMAGGCGATCGCCCTGTA
H.cetorum           AGAATAATGGCACAAAATCTTTGCATGTGGGTAGCCATTTTCATTTCTTTGAAGCTAATAGAGCCTTAGAATTTG
H.cynogastricus     ---------------------------------CACACTTCCACTTCTTTGAAGTCAACAAACTCTTGGATTTTG
H.acinonychis       AAAATGTGGGCGATAGACCCGTTCAAATCGGCTCTCACTTCCATTTCTTTGAAGTGAATAGATGCTTAGACTTTG
H.bizzozeronii      ATAACAAAGGCGATCGCCCCGTGCAAGTGGGCTCTCACTTCCACTTCTTTGAAGTTAATAAGCTTTTGGAATTTG
H.suis              AAAACACAGGCGATCGCCCTGTACAAGTAGGCTCTCACTTCCATTTCTTTGAAGTTAACAAACTGTTAGACTTTG
H.ailurogastricus   AAAACAAAGGCGATCGCCCTGTGCAAGTGGGCTCACACTTCCACTTCTTCGAAGTCAATAAGCTTCTAGACTTCG
H.heilmannii        AAAACAAAGGCGATCGCCCCGTGCAAGTGGGTTCACACTTCCACTTCTTCGAAGTCAACAAACTGCTCGACTTCG
H.baculiformis      AAAACAAAGGCGATCGCCCCGTGCAGGTGGGTTCACACTTCCATTTCTTTGAAGTTAACAAACTTTTAGATTTTG
H.salomonis         AAAACAAAGGTGATCGTCCTGTGCAGGTGGGATCACACTTCCATTTCTTTGAAGTCAACAAACTCTTGAACTTCG
H.felis             AAAATAAAGGCGATCGTCCTGTGCAGGTGGGATCACATTTCCACTTCTTCGAAGTGAATAAGCTCTTGGACTTCG
H.aurati            TCAATAAAGCAGACAGACCTATACAAGTAGGTTCGCATTTTCACTTTTTTGAAGTCAATAAATTATTGCAATTTG
H.didelphidarum     CAAATAAAGGCGATAGACCAATTCAGGTTGGTAGTCATTTTCATTTTTTTGAAGTAAATAAAAAACTTGTTTTTG
H.hepaticus         CTCATAAGGGCGATAGACCTATACAAGTAGGTTCACATTTTCATTTTTTTGAAGTCAATGCACTTTTGGAATTTG
H.bilis             CGAATAAGGGCGATAGACCTATACAAGTAGGCTCACATTTTCACTTTTTTGAGACAAATAAGCTTTTGGTATTTG
H.mustelae          GCAACAAAGGTGATAGACCTATTCAAGTGGGTTCACACTTCCATTTCTTTGAGACCAATAAACTGCTTGAGTTTG
H.anseris           ---------GCGATAGACCYATACAAGTRGGTTCCCACTTTCATTTTTTTGAAACTAATAAACTTTTAGAGTTTG
                                                       ** ** ** ** ** **     **         *    ** *
H.cetorum           ACAGAGAAAAAGCCTATGGCAGACGCTTAGATATTCCAAGCGGAAATACCTTAAGAATTGGAGCTGGTGAGACTA
H.cynogastricus     ATCGTGAAAAAGCCTATGGTAAACGCTTGGACATCGCCTCTGGTACTGCTGTGCGCTTTGAACCTGGTGAGACAA
H.acinonychis       ACAGAGAAAAAGCTTTCGGTAAGCGATTAGATATTGCGAGTGGGACAGCGGTAAGGTTTGAGCCTGGCGAAGAAA
H.bizzozeronii      ATCGTGAAAAAGCCTATGGCAAACGCCTAGACATTGCTTCTGGAACCGCTGTGCGCTTTGAACCCGGTGAGAAA-
H.suis              ATCGCGAAAAAGCTTATGGCAAACGACTTGACATCGCCTCTGGTACAGCTGTGCGCTTTGAACCTGGCGAAGAAA
H.ailurogastricus   ATCGCGAAAAAGCCTATGGCAAACGCCTAGACATCGCTTCAGGTACTGCTGTGCGCTTTGAACCCGGGGAAGAAA
H.heilmannii        ATCGTGAAAAAGCCTATGGTAAACGCTTGGACATTGCCTCTGGTACAGCCGTGCGCTTTGAACCTGGTGAAGAAA
H.baculiformis      ATCGTGAAAAAGCTTTCGGTAAGCGCTTAGACATCGCTTCAGGAACAGCTGTACGCTTTGAACCCGGAGAAGAAA
H.salomonis         ATCGCGAAAAAGCTTTTGGCAAACGCCTAGACATCGCTTCAGGGACAGCTGTGCGCTTTGAACCGGGTGAAAAAA
H.felis             ATCGCGCAAAAGCTTTTGGCAAACGCCTAGACATTGCATCTGGAACAGCGGTGCGCTTTGAACCCGGGGAGGAAA
H.aurati            ACAGAAAAAAAGGCATTGGGAAAACGTCTAGACATACCTTCAGGAACAGCCGTGAGATTCGAACCAGGCGAACGTA
H.didelphidarum     ATAGAGAACAAGCTTTTGGAAAACGATTAGACATAGCATCTGGAACTTCTGTGCGTTTTGAACCTGGGGAAAGTA
H.hepaticus         ATAGAGCCCAAGCATTTGGTAAACGTTTAGATATTGCCTCTGGCACATCAGTGCGTTTTGAGCCGGGTGAAGAAA
H.bilis             ATAGAGAGATGGCTTATGGCAAAAGGCTTGATATTGCCTCTGGGACTTCTGTGAGATTTGAGCCCGGAGAGACTA
H.mustelae          ATAGAGAAAAAGCTTATGGCACAAGACTTGACATTGCTTCAGGAACATCTGTGAGATTTGAACCAGGTGAGAGCA
H.anseris           ATAGACAAAAAGCCTATGGAAAAAGATTGGATATTGCATCTGGAACTTCTGTTAGATTTGAGCCAGGAGAAAGCA
                    *   *      ** *  ** *   *  * ** **  *    ** *   *  *  *  * *   * ** **
                                          ACGACTTGACATCGCCTCTGGTACAGC    CGCTTTGAACCTGGCGAAGAAA
                                          Probe (ureA_Hsuis)             Reverse primer (BF_HsuisR1)
```

Supplementary Figure S1. Nucleotide sequence alignment of *ureA* gene target, primers and probe, with mismatches marked in underlined bold. Accession numbers were for *H. cetorum* (CP003481), *H. cynogastricus* (EF070340), *H. acinonychis* (AM260522.1), *H. bizzozeronii* (FR871757), *H. suis* (AP023046), *H. ailurogastricus* (AP026687), *H. heilmannii* (AP026684), *H. baculiformis* (EF070343), *H. salomonis* (AF508005), *H. felis* (FQ670179), *H. aurati* (NXLW01000005), *H. didelphidarum* (NXLQ01000006), *H. hepaticus* (AE017125), *H. bilis* (CP019645), *H. mustelae* (L33462), *H. anseris* (KT599910).
